# SEOM Clinical Guideline update for the prevention of chemotherapy-induced nausea and vomiting (2016)

**DOI:** 10.1007/s12094-016-1583-0

**Published:** 2016-11-28

**Authors:** R. de las Peñas, A. Blasco, J. De Castro, Y. Escobar, R. García-Campelo, A. Gúrpide, R. Lopez-Lopez, M. Majem, C. A. Rodríguez, J. A. Virizuela

**Affiliations:** 1 Servicio de Oncología Médica, Consorcio Hospital Provincial de Castellón, Avda. Dr. Clará, 19, 12002 Castellón de la Plana, Castellón Spain; 2 Servicio de Oncología Médica, Hospital General Universitario de Valencia, Valencia, Spain; 3 Servicio de Oncología Médica, Hospital Universitario la Paz de Madrid, Madrid, Spain; 4 Servicio de Oncología Médica, Hospital General Universitario Gregorio Marañón de Madrid, Madrid, Spain; 5 Servicio de Oncología Médica, Complexo Hospitalario Universitario A Coruña (Hospital Juan Canalejo), A Coruña, Spain; 6 Servicio de Oncología Médica, Clínica Universitaria de Navarra, Pamplona, Spain; 7 Servicio de Oncología Médica, Complejo Hospitalario Universitario de Santiago de Compostela, Santiago de Compostela, Spain; 8 Servicio de Oncología Médica, Hospital de la Santa Creu I Sant Pau de Barcelona, Barcelona, Spain; 9 Servicio de Oncología Médica, Hospital Universitario de Salamanca, Salamanca, Spain; 10 Servicio de Oncología Médica, Complejo Hospitalario Regional Virgen Macarena de Sevilla, Sevilla, Spain

**Keywords:** Chemotherapy-induced emesis, Antiemetic drugs, Prophylaxis of emesis, SEOM guidelines of antiemetic drugs

## Abstract

Chemotherapy-induced nausea and vomiting is one of the most worrisome adverse effects of chemotherapy for cancer patients. It can cause severe discomfort and affect the quality of life. In recent years, the incorporation of new drugs has increased the efficacy of antiemetic treatments in the control of emesis associated with chemotherapy. This guideline, in which we give some treatment recommendations with level of evidence and grade of recommendation, provides an update of the previously published guideline of the Spanish Society of Medical Oncology and represents our continued commitment to improving supportive care in cancer patients.

## Introduction

Chemotherapy-induced nausea and vomiting (CINV) is one of the most worrisome adverse effects of chemotherapy for cancer patients. It can cause severe discomfort and affects quality of life. The probability of suffering from chemotherapy-induced nausea and vomiting depends on several factors, some of which are directly related to the drugs used and others that are patient-dependent [1, 2]. Certain patient characteristics increase the frequency of emesis with anti-cancer treatments: poor general condition, being young, female, low or no alcohol consumption, the presence of emesis in previous chemotherapy treatments, anticipatory emesis, and psychological disorders, such as anxiety. Likewise, dehydration and metabolic disorders such as hyperkalemia, concurrent treatments (opioids, antibiotics, antifungals, etc.), and a history of motion sickness or hyperemesis gravidarum may increase the likelihood of emesis. Furthermore, certain polymorphisms of the enzymes that metabolize 5-HT3 receptor antagonists and of the receptor itself are associated with a greater risk of emesis [3]. Knowing these factors makes it possible to adapt antiemetic treatment to each patient, especially in the presence of more than one of the aforenamed factors.

The classification of emetogenic potential of cytostatics encompasses four categories: high, moderate, low, and minimal. However, it must be noted that a significant number of patients receive treatments consisting of a combination of several cytostatic drugs instead of monotherapy. One of the issues to be resolved is how to gauge the emetogenic potential of the said combinations (Table 1).

**Table 1:** Emetogenic potential of cytostatics and their combinations

Highly emetogenic chemotherapy (>90% of patients vomit). Level 4
Cisplatin, mechlorethamine, streptozotocin, cyclophosphamide >1500 mg/m^2^
Carmustine, dacarbazine
Oral cytostatics:
Hexamethylmelamine, procarbazine
Moderately emetogenic chemotherapy (30–90% of patients vomit). Level 3
Oxaliplatin, cytarabine (>1 g/m^2^)
Carboplatin, ifosfamide, cyclophosphamide <1500 mg/m^2^
Anthracyclines, irinotecan
Oral cytostatics:
Cyclophosphamide, etoposide, temozolomide, vinorelbine, imatinib
Low emetogenic chemotherapy (10–30% of patients vomit). Level 2
Taxanes, mitoxantrone, topotecan, etoposide, pemetrexed, methotrexate, mitomycin C, gemcitabine, cytarabine, 5-Fu, bortezomib, cetuximab, trastuzumab
Oral cytostatics:
Capecitabine, fludarabine
Minimally emetogenic chemotherapy (<10% of patients vomit). Level 1
Bleomycin, busulfan, 2-clorodeoxiadenosin, fludarabine, vinca alkaloids, bevacizumab
Oral cytostatics:
Chlorambucil, hydroxyurea, methotrexate, gefitinib
Emetogenic potential of combinations
Determined by the drug with the highest emetogenic potential
The use of drugs in level 3 increases emetogenic level of the combination (FAC, FEC, AC, TAC, etc)
The use of drugs in levels 1 and 2 does not change the emetogenic level of the combination

Modified of Hesketh, Grunberg and García-Gómez [4–6]

The recent years have witnessed the introduction of new molecules that have improved the control of chemotherapy-induced emesis. For this reason, the Spanish Society of Medical Oncology (SEOM) believes that the time has come to review and update the previous Clinical Guidelines published in 2010 [6] to include new developments.

## Guideline methods

Under the auspices of the Spanish Society of Medical Oncology (SEOM), a number of experts in the field together with two coordinators were designated to develop these evidence-based, clinical practice guidelines. Recommendations and evidence have been graded, based on the guideline development recommendations [7].

## Diagnosis

Four types of CINV can be defined: acute, delayed, anticipatory, and incidental episodic emesis [8].

- Acute emesis occurs within the first 24 h following chemotherapy infusion, most often between 2 and 6 h post-infusion.
- Delayed emesis occurs 24 h after chemotherapy is administered. It most typically ensues between 48 and 72 h and is generally associated with drugs such as cisplatin, carboplatin, cyclophosphamide, and anthracyclines.
- Anticipatory emesis develops during the hours prior to receiving cytostatic treatment.
- Incidental episodic emesis appears more than 120 h after receiving chemotherapy.

## Treatment

The treatment of chemotherapy-associated emesis is based on drugs that inhibit or antagonize signaling of some of the neurotransmitters involved in the process. The drugs used in antiemetic prophylaxis can be divided as:

- The classical antiemetic agents, prior to the 1990s, are gradually losing relevance, although they can still be very useful in specific situations, such as refractory emesis, or when modern agents or steroids are contraindicated. These drugs are dopaminergic receptor (subtype D2) antagonists and include phenothiazine (proclorpromacine, perphenazine, and tietilperacilin), butyrophenones, (haloperidol and droperidol), and substituted benzamides (metoclopramide, domperidone, and alizapride) [9].
- Introduced in the early 1990s, competitive serotonergic receptor (5-Hydroxytryptamine-3 or 5-HT 3 subtype) antagonists are the reference antiemetic drugs since then. First-generation 5-HT3 receptor antagonists include ondansetron, granisetron, dolasetron, tropisetron, and second-generation agents in this class include palonosetron. First-generation drugs have similar efficacy that increases when administered with steroids. Ondansetron should not be used in patients with congenital prolonged QT-interval syndrome and should be monitored in patients with electrolyte abnormalities, congestive heart failure, bradyarrhythmias or when other drugs that may prolong the QT interval are administered. Palonosetron has demonstrated greater efficacy than first-generation setrons in phase III trials; it produces a long-lasting serotonin receptor blockade, and has synergistic activity with neurokinin inhibitors [10].
- Substance P antagonists (of the neurokinine-1 receptor, NK-1): aprepitant, fosaprepitant, netupitant, rolapitant. In combination with 5-HT3 receptor antagonists and steroids, they offer better control in acute and delayed emesis in highly emetogenic chemotherapy regimens [11, 12].
- Steroids: dexamethasone, methylprednisolone. Their mechanism of action is unknown, although it is probably related to the inhibition of prostaglandin synthesis and there is ample scientific evidence that supports their use. The most widely used drug in this class is dexamethasone, which offers great efficacy when used either in monotherapy or in combination.
- Benzodiazepines: lorazepam, alprazolam, midazolam, and olanzapine (a thienobenzodiazepine with antipsychotic effects, which enhances the antiemetic effect, especially in delayed nausea [13 **]**.
- Cannabinoids (dronabinol, nabilone): They can provide an antiemetic effect in patients who do not respond to 5-HT3 receptor antagonists and NK-1 receptors and in the prevention of anticipatory emesis.

The combination of the different drugs, as stated in the guidelines, allows for antiemetic therapy to be adapted to each patient and clinical situation:

1. Prophylactic antiemetics in treatments with high emetogenic potential [12–14]:
  Highly emetogenic chemotherapy is considered to be those agents or schedules that would cause vomiting in more than 90% of the cases in the absence of antiemetic prophylaxis.
  - HEC prophylaxis consists of administering a triplet containing 5-HT3 receptor antagonists, neurokinine-1 receptor inhibitors, and steroids. (Level of Evidence I, Grade of Recommendation A).
    (i) Of the 5-HT3 receptor antagonists, 0.25 mg of palonosetron has proven to be the most efficacious (Level of Evidence II, Grade of Recommendation B). Other agents that have been used are granisetron (0.01 mg/kg (max. 1 mg) IV or 2 mg *per os*) and ondansetron (8–16 mg IV or 16–24 mg orally). Administration of these drugs after chemotherapy is not recommended because it has not proven to be beneficial and they have associated side effects.
    (ii) There are currently several neurokinine-1 receptor inhibitors to choose from: aprepitant (orally at a dose of 125 mg on day 1 and 80 mg on days 2 and 3) or fosaprepitant (150 mg iv) on day 1. Netupitant is a second-generation NK1 receptor antagonist that targets the serotonin and substance P-mediated pathways involved predominantly in delayed emesis. Oral netupitant is combined with oral palonosetron (NEPA) in a single tablet. NEPA is approved for the prevention of CINV in patients receiving HEC and MEC based on 3 clinical trials. (Level of Evidence IB, Grade of Recommendation A).
    (iii) Efficacy of Olanzapine + palonosetron + dexamethasone in terms of CR rates did not differ significantly compared to aprepitant + palonosetron + dexamethasone. (Level of Evidence IB, Grade of Recommendation A).
    (iv) Steroids: Dexamethasone is administered PO/IV at varying doses depending on the schedule used (Table 2).

**Table 2:** Prophylactic antiemetic treatment with high emetogenic potential

Day 1	Day 2	Day 3	Day 4
Option A			
5HT-3 receptor antagonist^a^			
Dexamethasone 12 mg PO/IV	Dexamethasone 8 mg PO/IV daily	Dexamethasone 8 mg PO/IV daily	Dexamethasone 8 mg PO/IV daily
Aprepitant or Fosaprepitant	Aprepitant	Aprepitant	
Option B			
NEPA = Netupitant 300 mg/palonosetron 0.5 mg PO			
Dexamethasone 12 mg PO/IV	Dexamethasone 8 mg PO/IV daily	Dexamethasone 8 mg PO/IV daily	Dexamethasone 8 mg PO/IV daily
Option C			
Olanzapine 10 mg PO	Olanzapine 10 mg PO daily	Olanzapine 10 mg PO daily	Olanzapine 10 mg PO daily
Palonosetron 0.25 mg IV			
Dexamethasone 20 mg PO/IV			

^a^Palonosetron is advised because of its superiority in controlling delayed emesis
2. Prophylactic antiemetics in treatments with moderate emetogenic potential [15–18] (Table 3):
  Drugs of moderate emetogenic potential are those with an associated risk of emesis of between 30 and 90%.
  - A 5-HT3 receptor antagonist is recommended together with dexamethasone for acute emesis. (Level of Evidence II, Grade of Recommendation B). The anti-5-HT3 of choice is palonosetron.
  - As alternatives: Anti-5-HT3 + DEX ± Anti-NK1; NEPA + D
  - Dexamethasone; olanzapine + palonosetron + dexamethasone. (Level of Evidence II, Grade of Recommendation B).
  - For carboplatin-containing chemotherapy, some guidelines recommend the combination of a NK1 receptor antagonist with a 5-HT3 and dexamethasone. The anti-NK1 can be aprepitant, fosaprepitant, or NEPA administered on day 1. If aprepitant (125 mg on day 1) has been used, it is recommended that it also be administered on days 2 and 3, at a dose of 80 mg to prevent delayed emesis. If other NK1 receptor antagonists have been used on day 1, no further antiemesis is needed. (Level of Evidence II, Grade of Recommendation B).
  - Routine profilaxis for delayed emesis cannot be recommended for the majority of patients receiving QME. (Level of Evidence IV, Grade of Recommendation D). However, in patients who receive QME known to potentially cause delayed emesis (oxaliplatin, anthracyclines, or cyclophosphamide), the use of dexamethasone on days 2 and 3 should be considered. (Level of Evidence III, Grade of Recommendation C). Alternatively: anti 5-HT3 on days 2 and 3 + olanzapine on days 2 and 3. (Level of Evidence II, Grade of Recommendation B).
3. Prophylactic antiemetics in treatments with low and minimal emetogenic potential [19]:
  Drugs associated with low emetic potential are those for which the risk of emesis lies between 10 and 30%. For drugs having a minimal emetic potential, the risk is <10%. Most new-targeted agents are included in this category.
  - The optimal treatment to prevent nausea and vomiting due to low emetogenic antineoplastic agents includes a single antiemetic like dexamethasone, a dopamine receptor antagonist, such as metoclopramide, or 5-HT3 receptor antagonist (Level of Evidence II, Grade of Recommendation B).
  - No antiemetic should be administered routinely before or after chemotherapy from minimally emetogenic antineoplastic agents to patients without a history of nausea and vomiting (Level of Evidence IV, Grade of Recommendation D).
  - If a patient experiences nausea or vomiting, preventive antiemetic treatment might be considered for subsequent chemotherapy treatments using the regimen for the next higher emetic level.
4. Prevention of nausea and vomiting induced by multiple-day chemotherapy, refractory nausea and vomiting and rescue antiemetic therapy [10]:
  - Prophylaxis in patients receiving moderately- or highly-emetogenic multiday chemotherapy is more difficult, due to a mixture of acute and delayed effects, as well as anticipatory emesis. Practical issues should be considered (i.e. route of administration, duration of action of 5-HT3 receptor antagonists, dosing intervals, or individual risk factors). Moreover, there are few clinical studies that look at this situation.
    (i) Patients receiving multiple-day moderately or highly emetogenic chemotherapy should receive a 5-HT3 receptor antagonist plus dexamethasone for acute nausea and vomiting, and dexamethasone for delayed nausea and vomiting (Level of Evidence II, Grade of Recommendation A).
    (ii) If the regimen does not contain a NK1 receptor antagonist, the preferred serotonin antagonist is palonosetron.
    (iii) A NK1 receptor antagonist may be added in highly emetogenic chemotherapy.
  - Refractory nausea and vomiting and rescue antiemetic therapy is a challenging situation. Other causes for emesis (i.e. use of opiates, central nervous system metastases, hypercalcemia, or gastrointestinal obstruction) must be ruled out. Patients must have received appropriate antiemetic treatment.
    (i) The general principle is to adjust the regimen to be used for a higher risk group. Some patients may require new agents with different mechanisms of action (i.e. lorazepam, alprazolam, olanzapine, prochlorperazine, or haloperidol). Olanzapine has shown superiority over metroclopramide in a recent randomized trial.
5. Prevention of anticipatory nausea and vomiting [20]:
  Anticipatory nausea and vomiting is usually due to a learned response to chemotherapy, and increases with each subsequent cycle. Many individual factors such as age <50 years, anxiety, nausea, and vomiting after the previous chemotherapy treatment may predict the appearance of anticipatory nausea and vomiting.
  - The best approach to prevent anticipatory nausea and vomiting is the best possible control of acute and delayed nausea and vomiting (Level of Evidence III, Grade of Recommendation).
  - Behavioral therapies and hypnosis may be used to treat anticipatory nausea and vomiting (Level of Evidence II, Grade of Recommendation B).
  - Benzodiazepines can reduce the occurrence of anticipatory nausea and vomiting, largely by decreasing the anxiety generated by the administration of chemotherapy. The drug most commonly used is lorazepam (Level of Evidence II, Grade of Recommendation A).
6. Prevention of radiotherapy-induced nausea and vomiting [21]:
  The emetic risk of radiotherapy is divided into four risk levels: high, moderate, low, and minimal. These levels depend on the site of radiation and do not take into account radiation dose, fractionation, or technique, or other proposed risk factors.
  - Patients receiving highly emetic radiation therapy (total body irradiation) should receive a 5-HT3 receptor antagonist plus dexamethasone (Level of Evidence II, Grade of Recommendation B).
  - Those receiving moderately emetic radiation therapy (upper abdomen, craniospinal locations) should receive a 5-HT3 receptor antagonist and optional short-course dexamethasone (Level of Evidence II, Grade of recommendation A).
  - Subjects who are given low emetic radiation therapy (thorax, cranium, head and neck, and pelvis) should receive prophylaxis or rescue with a 5-HT3 receptor antagonist, dexamethasone, or a dopamine receptor antagonist (Level of Evidence IV, Grade of Recommendation D).

Patients receiving minimally emetic radiation therapy (extremities, breast) should receive rescue treatment with a dopamine receptor-antagonist or a 5-HT3 receptor antagonist. (Level of Evidence IV, Grade of Recommendation D).

**Table 3:** Prophylactic antiemetic treatment with moderate emetogenic potential

Day 1	Day 2	Day 3
5HT-3 receptor antagonist^a^		
Dexamethasone 12 mg PO/IV	Dexamethasone 8 mg PO daily	Dexamethasone 8 mg PO daily

See text for alternative schemes

^a^Palonosetron is advised because of its superiority
